# *Operando* deconvolution of photovoltaic and electrocatalytic performance in ALD TiO_2_ protected water splitting photocathodes†
†Electronic supplementary information (ESI) available: SEM images, Faradaic efficiencies, *V*2/Δ*V*–*V*1 curve, *etc.* See DOI: 10.1039/c8sc01453a


**DOI:** 10.1039/c8sc01453a

**Published:** 2018-06-05

**Authors:** Wei Cui, Wenzhe Niu, René Wick-Joliat, Thomas Moehl, S. David Tilley

**Affiliations:** a Department of Chemistry , University of Zurich , Winterthurerstrasse 190 , CH-8057 Zurich , Switzerland . Email: david.tilley@chem.uzh.ch; b State Key Laboratory of Silicon Materials , School of Materials Science and Engineering , Zhejiang University , Hangzhou , Zhejiang , China

## Abstract

In this work, we demonstrate that buried junction photocathodes featuring an ALD TiO_2_ protective overlayer can be readily characterized using a variation of the dual working electrode (DWE) technique, where the second working electrode (WE2) is spatially isolated from the hydrogen-evolving active area. The measurement of the surface potential during operation enables the *operando* deconvolution of the photovoltaic and electrocatalytic performance of these photocathodes, by reconstructing *J*–Δ*V* curves (reminiscent of photovoltaic *J*–*V* curves) from the 3-electrode water splitting data. Our method provides a clearer understanding of the photocathode degradation mechanism during stability tests, including loss of the catalyst from the surface, which is only possible in our isolated WE2 configuration. A pn^+^Si/TiO_2_ photocathode was first investigated as a well behaved model system, and then the technique was applied to an emerging material system based on Cu_2_O/Ga_2_O_3_, where we uncovered an intrinsic instability of the Cu_2_O/Ga_2_O_3_ junction (loss of photovoltage) during long term stability measurements.

## Abstract 2

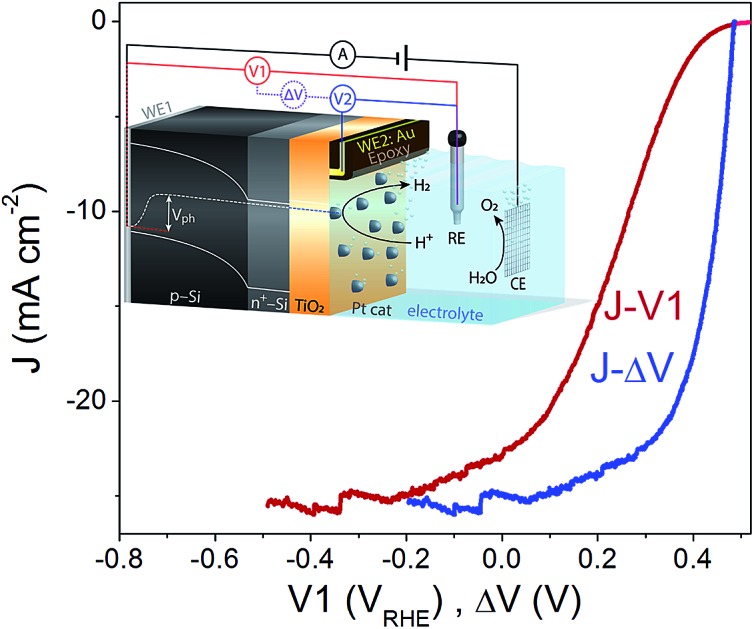
The dual-working-electrode technique enables the deconvolution of the intrinsic properties of the buried p–n junction and the electrocatalyst on the surface for water splitting photocathodes.

## Introduction

Photoelectrochemical (PEC) water splitting has been recognized as a promising avenue for harvesting renewable hydrogen fuel from inexhaustible solar energy and water.^1^–^4^ Large photovoltages are required for efficient and therefore cost-effective water splitting, and one approach to achieving larger open-circuit voltages (*V*_oc_) is using so-called “buried junctions”. These buried junction photoelectrodes can be modeled as a series combination of a p–n junction photoabsorber, a protective layer and surface catalyst (pn/cat),^5^,^6^ where the *V*_oc_ is decoupled from the semiconductor–electrolyte interface, and the increased band bending of the p–n junction can significantly enhance electron–hole pair separation.^7^

The efficiency of a pn/cat photocathode is largely determined by the intrinsic properties of the buried p–n junction.^8^ However, the semiconductor-catalyst and catalyst–electrolyte interfaces also play a critical role in the overall performance of the system. Issues such as charge transport in the protective layer, the nature of the semiconductor-catalyst contact (ohmic or Schottky-type), as well as the electrocatalytic activity at the catalyst–electrolyte interface are typically obscured within the standard current–voltage measurement data.^9^,^10^ Therefore, we sought to develop an experimental technique that could not only evaluate the PEC performance but also simultaneously provide an understanding of these different interfaces during PEC operation.

The dual working electrode (DWE) technique was first reported in the 1970s. Nakato, Pinson and Wilson reported that n-GaP and n-TiO_2_ photoanodes coated with thin gold films showed a photovoltaic effect, representing early examples of *in situ* measurements of the surface potential.^11^–^13^ Recently, the Boettcher group has used the DWE technique to study a photoanode-catalyst interface.^14^ It is of note that the second working electrode in all of the previous works has either been a transparent conducting oxide (TCO) or a thin metal film that covers the entire active area.^15^,^16^ For systems that do not employ TCOs as part of the buried junction structure, it has thus far not been possible to carry out DWE studies without introducing a metallic film, which influences the measurement through partial light absorption and by affecting the catalyst binding to the photoelectrode surface. We have therefore developed a new architecture of the DWE technique that is compatible with standard buried junction photocathodes featuring a protective layer, which does not introduce extraneous materials at the semiconductor–electrolyte interface. With this method, one can diagnose a problem of the stability of the catalyst on the surface *versus* the stability of the photovoltaic output of the p–n junction. As will be shown in this manuscript, the latter case does indeed require consideration. The diagnosis of the point of failure in unmodified PEC devices under operation is critical for identification of targets to improve the system.

A TiO_2_-protected pn^+^-Si junction photocathode was chosen as a platform to develop this method, as the Si p–n junction is robust and stable. Atomic layer deposition (ALD) TiO_2_ is a common protective layer for water splitting photocathodes due to its favorable conduction band position for the hydrogen evolution reaction, optical transmittance for visible light, high stability over a wide range of electrolyte solutions and pH, and good conductivity. Moreover, the high doping density of ALD TiO_2_ enables an ohmic (tunnel) contact to the contacting metal of the second working electrode (WE2), no matter the work function.^17^,^18^ The top contact was made *via* a thin Au layer covered by epoxy, which was able to sense the surface potential of the photocathode under *operando* conditions, without directly contacting the electrolyte or HER catalyst. The hidden *J*–*V* curve of the buried p–n junction can then be extracted by measuring the difference in voltage between the backside and the surface of the photocathode (Δ*V*) and plotting *versus* the water splitting current. By monitoring the evolution of the hidden *J*–*V* curve in the 3-electrode water splitting measurements, one can immediately diagnose whether the degradation in the performance of the photocathode derives from a problem with the catalyst or with the photovoltaic output of the p–n junction. In this work, we evaluate both a well understood system (pn^+^-Si) and a promising emerging system (p–n Cu_2_O/Ga_2_O_3_). The results from the Si system demonstrate that the failure of the surface catalyst is easily identified with our DWE technique. The results from the Cu_2_O-based system reveal an intrinsic instability of the p–n junction, with reduced photovoltaic output following a stability measurement.

## Results and discussion

### Interface energetics of the pn^+^Si/TiO_2_/Pt(ed) photocathode

**Fig. 1:**
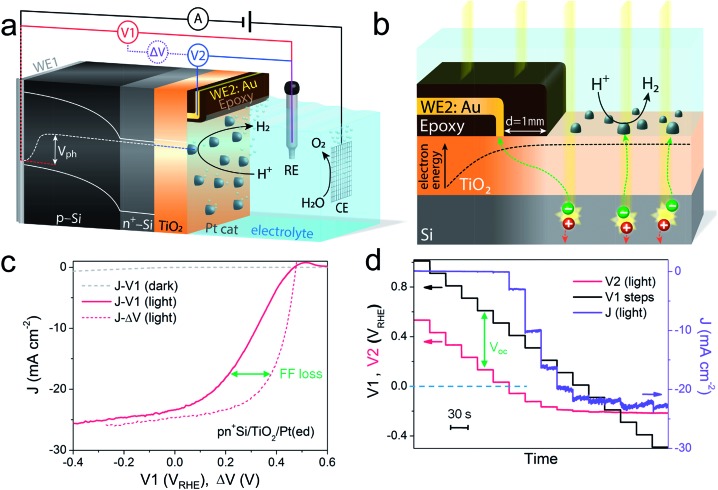
Schematic illustration of (a) the DWE configuration used during PEC measurements with a pn^+^Si/TiO_2_/Pt photocathode and (b) the structure of the sensing electrode WE2, located a small distance (∼1 mm) away from the illuminated area, separated by a thin coating of opaque epoxy (not to scale). For simplicity, the band bendings at the interfaces of the highly doped n^+^-Si and TiO_2_ have been omitted. (c) The *J*–*V*1 and *J*–Δ*V* curves of pn^+^Si/TiO_2_/Pt(ed), collected by a LSV scan toward negative potential with a scan rate of 10 mV s^–1^ in 0.5 M H_2_SO_4_. Δ*V* = *V*1 – *V*2. (d) *V*2 and *J* values of pn^+^Si/TiO_2_/Pt(ed) with stepwise controlled *V*1 under illumination. Each *V*1 step lasts 30 s. Pt(ed) indicates that the Pt was deposited by electrodeposition.

A pn^+^-Si photocathode with 100 nm-thick ALD-TiO_2_ protective layer was chosen as a model system for the development of the technique. A schematic diagram of the DWE setup is depicted in Fig. 1a. WE1 is used for controlling the back contact potential (*V*1) of the photocathode. WE2 is connected to the photocathode surface and kept at open circuit during PEC measurements to directly probe the surface potential (or in other words to probe the energy level of surface-accumulated electrons) in relation to the reference electrode (*V*2). To avoid direct contact of WE2 and the active area, WE2 was contacted a small distance away from the illuminated area (∼1 mm) and separated by a thin coating of opaque epoxy, as shown in Fig. 1b. When illuminated, electron–hole pairs are generated and separated by the built-in electric field across the p–n junction, bringing photoelectrons to the surface while sweeping holes to the back contact. Consequently, a photovoltage is created across the pn^+^-Si homojunction. Considering that both the n-type Si layer and TiO_2_ are highly doped,^19^ the width of the space charge region of both n^+^-Si and TiO_2_ is very narrow and ensures an Ohmic contact. The Pt catalyst also forms an Ohmic contact with the TiO_2_ layer.^17^ As a result, the measured potentials of *V*1 and *V*2 directly give the energetic positions of the quasi-Fermi level of holes and electrons, respectively. The difference between *V*1 and *V*2, denoted Δ*V*, is the output voltage of the pn^+^-Si junction. It is worth noting that as the photoelectrons diffuse away from the illuminated area (as shown in Fig. 1b), the electrons cannot enter the electrolyte and will ultimately recombine. This leads to a lower electron density than in the illuminated area and a slightly reduced *V*_oc_ compared to the photovoltaic output of the p–n junction is recorded. The small drop in the measured surface potential is robust and reproducible, and does not complicate the analysis herein.

After the Pt catalyst was electrodeposited onto the TiO_2_ surface, the conventional current density-back contact potential (*J*–*V*1) curve of the pn^+^Si/TiO_2_/Pt(ed) photocathode was obtained in 0.5 M H_2_SO_4_ with a linear sweep from positive to negative potential (Fig. 1c). Under one sun illumination, the pn^+^Si/TiO_2_/Pt(ed) photocathode exhibits an onset potential for water reduction of ∼0.5 V_RHE_. As *V*1 becomes more negative, the photocurrent density increases and eventually saturates at 25 mA cm^–2^ at *V*1 = –0.2 V_RHE_. WE2 enables the *in situ* measurement of surface potential *V*2 during the sweep of *V*1. ESI Fig. S3† presents *V*2 and Δ*V* values as a function of *V*1 with and without illumination.

A hidden *J*–Δ*V* curve, analogous to the current–voltage characteristic of a PV cell, can then be extracted and is plotted in Fig. 1c. The *V*_oc_ and *J*_sc_ are 475 mV and 24.6 mA cm^–2^, respectively (the characteristics are also listed in ESI Table S1†). A significant loss of fill factor is observed when comparing the *J*–*V*1 and *J*–Δ*V* curves, which derives from the additional series resistances in a PEC cell *versus* a PV cell, namely the TiO_2_/catalyst junction resistance, the overpotential of the catalyst required for driving a chemical reaction, and the solution resistance.^20^ In essence, the *J*–Δ*V* curve shows the best possible fill factor that can be achieved by the *J*–*V*1 curve. In practice, a real PEC *J*–*V*1 curve will always have a smaller fill factor due to the catalyst overpotential as well as the series resistances mentioned above.

In order to more clearly visualize the effect of the surface potential on the current, a stepwise chronoamperometry measurement was carried out whereby the potential of *V*1 was stepped every 30 s and both *V*2 and the photocurrent were recorded (Fig. 1d). When *V*1 is positive of ∼0.5 V_RHE_ (and the photocurrent is still nearly 0), *V*2 remains at a constant distance (constant Δ*V*, see also ESI Fig. S3†). Substantial cathodic photocurrents appear at *V*1 = 0.4 V_RHE_, where *V*2 is (due to the photovoltage) more negative than 0 V_RHE_ (with Δ*V* now starting to shrink). After the onset potential, although *V*2 continues to move negatively with each step, Δ*V* shrinks further as the photocurrent increases. Ultimately, both *V*2 and the photocurrent become constant, even as *V*1 becomes more negative, eventually entering a reverse bias-type regime. ESI Fig. S4† depicts band energy diagrams under several conditions of applied bias, and ESI Fig. S5† gives a detailed discussion of the relationship between photovoltage and the onset potential.

### Stability of the pn^+^Si/TiO_2_/Pt(ed) photocathode

A critical issue for photoelectrodes is the long-term stability. A standard procedure for assessing the stability is to carry out a chronoamperometry experiment under a static back contact potential and to compare the *J*–*V*1 behavior before and after the stability test. This type of analysis, however, is relatively limited because the underlying degradation mechanisms are inaccessible. The decrease in PEC performance can be due to several factors. Firstly, the H_2_-evolving catalyst may be deactivated, poisoned or dislodged from the electrode surface. Secondly, the p–n junction may produce a reduced output *V*_oc_, due to partial photocorrosion and increased recombination. These changes in the semiconductor material also result in lower photocurrent densities and fill factor.

**Fig. 2:**
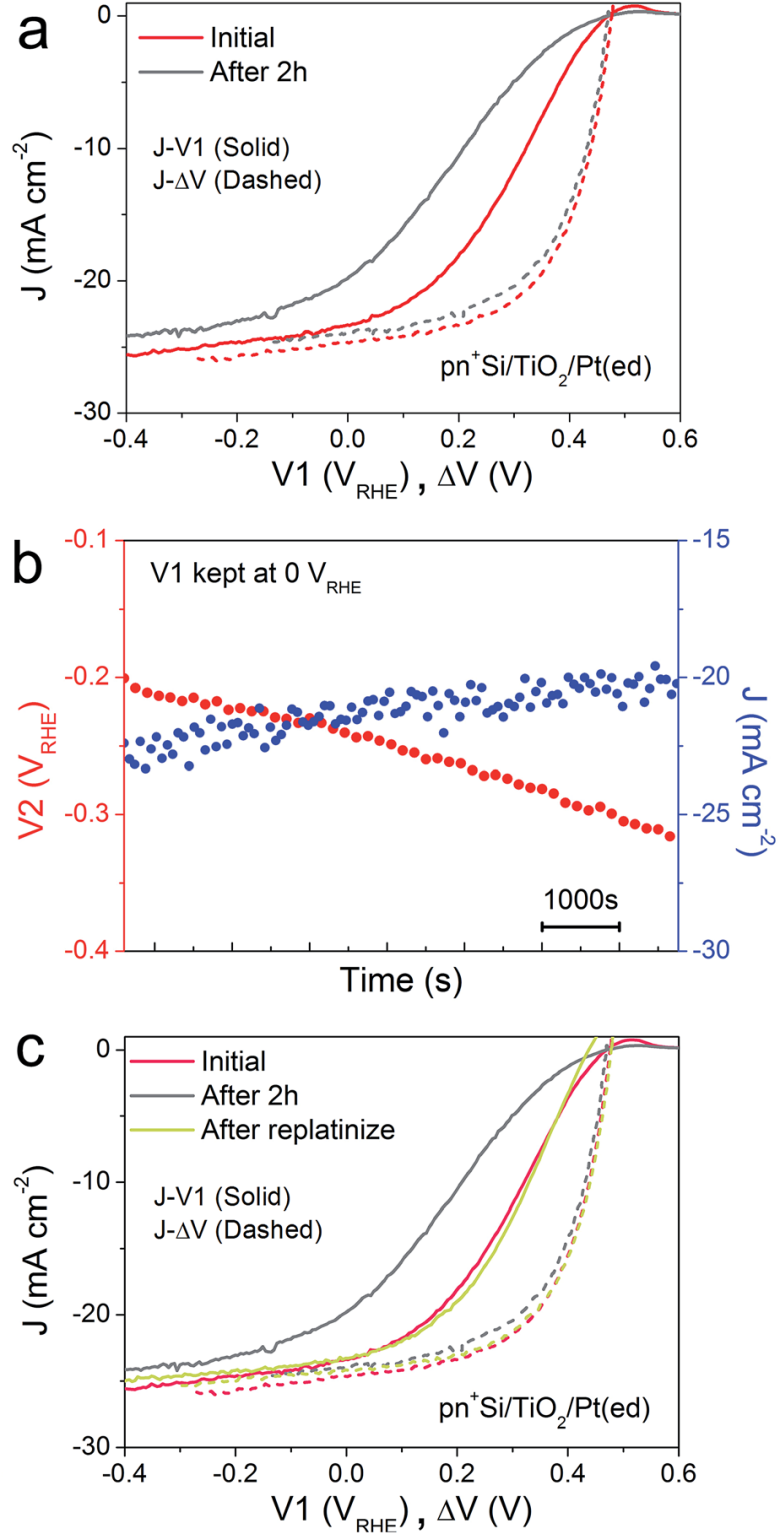
(a) *J*–*V*1 (solid) and *J*–Δ*V* curves (dashed) of pn^+^Si/TiO_2_/Pt(ed) before and after a 2 h stability test, collected by a LSV scan with a scan rate of 50 mV s^–1^ towards negative potential in 0.5 M H_2_SO_4_. (b) Changes in *V*2 and *J* during a 2 h stability test. *V*1 is held at 0 V_RHE_. (c) *J*–Δ*V* curves (dashed) of pn^+^Si/TiO_2_/Pt(ed) and the corresponding *J*–*V*1 curves (solid). All data are collected under simulated one sun illumination.

In order to characterize the degradation mechanism in the Si photocathode, we performed a 2 h stability test by holding *V*1 at 0 V_RHE_, a typical value for these types of test in the literature.^8^Fig. 2a shows the *J*–*V*1 and *J*–Δ*V* curves before and after the 2 h stability test. Compared with the initial *J*–*V*1 scan, the scan after the 2 h shows similar onset potential and slightly decreased saturation photocurrent, but a remarkably poorer fill factor. As the *J*–Δ*V* curves remain the same, it is immediately apparent that the problem relates to the catalyst and not to the photovoltaic performance of the buried junction. Fig. 2b depicts how the surface potential *V*2 and photocurrent density change over time under a static back contact potential of 0 V_RHE_. Over 2 h, the photocurrent density drops from ∼23 to ∼20 mA cm^–2^, while *V*2 steadily shifts to more negative values, which indicates that higher overpotential is needed in order to achieve a similar current density. A poor contact between the surface and the catalyst (TiO_2_/catalyst) as well as worsening kinetics at the catalyst/electrolyte interface (*e.g.* surface poisoning) will result in a higher overpotential for the catalytic interface.^21^ Pt was then re-deposited onto the electrode surface (Fig. 2c). Due to the fact that the fill factor is completely restored upon re-platinization, we can confirm that neither a degradation in the p–n junction of the silicon nor resistive losses through *e.g.* formation of a silicon oxide layer are responsible for the change in the *J*–*V*1 curve. The degradation likely results from desorption of the Pt nanoparticles, as has been previously observed for electrodeposited platinum on ALD TiO_2_.^22^ When the ALD TiO_2_ was replaced by a thin metallic Ti film, the Pt catalyst binding was much more robust over a 2 h stability measurement (ESI Fig. S7†)

### Investigation of the TiO_2_/Pt junction

**Fig. 3:**
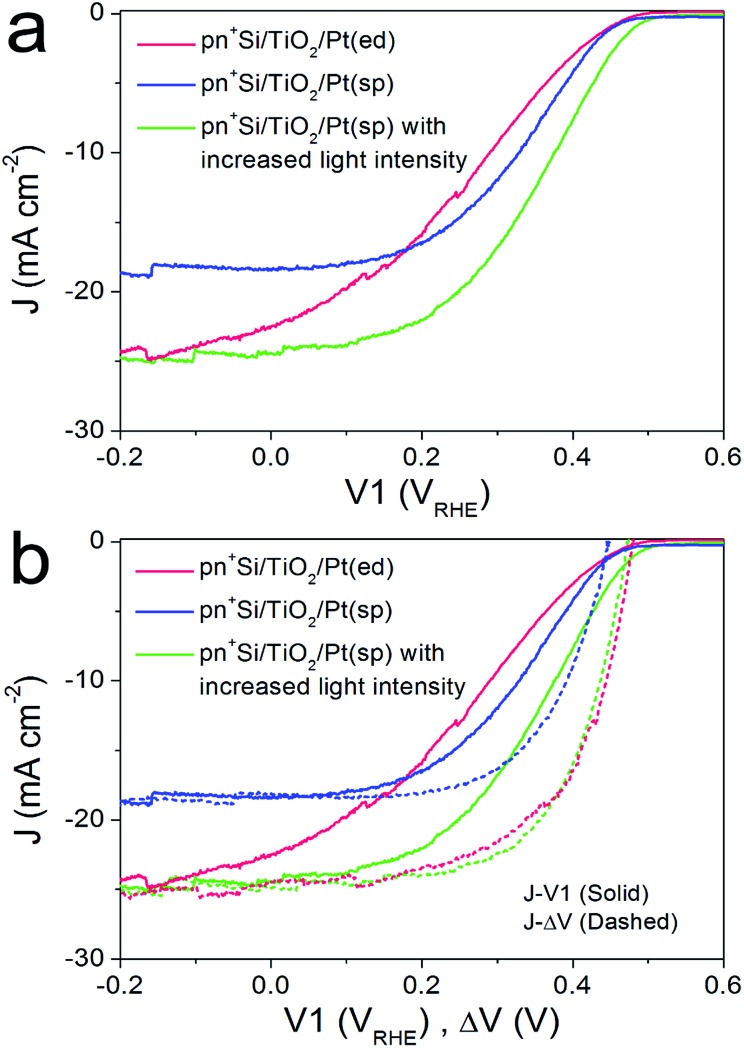
(a) Comparison of *J*–*V*1 curves between pn^+^Si/TiO_2_/Pt(ed) and pn^+^Si/TiO_2_/Pt(sp), collected by a LSV scan with a scan rate of 10 mV s^–1^ towards negative potential in 0.5 M H_2_SO_4_. (b) *J*–Δ*V* curves (dashed) of pn^+^Si/TiO_2_/Pt(ed) and pn^+^Si/TiO_2_/Pt(sp), combined with their *J*–*V*1 curves (solid). All data are collected under illumination. For comparison, the performance of pn^+^Si/TiO_2_/Pt(sp) with similar photocurrent densities as pn^+^Si/TiO_2_/Pt(ed), by increasing the light intensity, is also displayed (green solid and dashed curves).

For the pn^+^-Si photocathodes described above, the Pt catalyst was electrodeposited onto either the TiO_2_ or Ti metal surface as nanoparticles with a size range of ∼10–30 nm (ESI Fig. S8a†). This non-continuous catalyst morphology may be unfavorable for efficient extraction of the surface electrons, resulting in a poor fill factor of the *J*–*V*1 curve.^23^ Therefore, we investigated a nominally 2 nm-thick Pt film with nearly full coverage on the TiO_2_ layer by sputter coating (denoted pn^+^Si/TiO_2_/Pt(sp)). The Pt deposited in this way makes the surface slightly rough (ESI Fig. S8b†). Fig. 3a compares the *J*–*V*1 curves of pn^+^Si/TiO_2_/Pt(sp) and pn^+^Si/TiO_2_/Pt(ed) photocathodes under one sun illumination. Sputtered Pt exhibits a similar onset potential and improved fill factor, but much reduced photocurrent densities due to the optical transmission loss through the 2 nm-thick Pt film (ESI Fig S9†). For better comparison between the sputtered and electrodeposited samples, we also measured the *J*–*V*1 curve of the pn^+^Si/TiO_2_/Pt(sp) at an increased light intensity to achieve a similar photocurrent density, plotted in green. The green curve exhibits an earlier onset potential despite having the same *V*_oc_ as the Pt(ed) curve, suggesting a better catalytic activity of sputtered Pt over electrodeposited Pt. Support for this hypothesis is shown by comparing their individual catalytic activities towards H_2_ generation when deposited on FTO slides (Fig S10†). Additionally, pn^+^Si/TiO_2_/Pt(sp) always shows an enhancement in the fill factor the in *J*–*V*1 curves, reflecting the smaller TiO_2_/Pt/electrolyte interfacial resistance for the TiO_2_/Pt(sp) as compared to the TiO_2_/Pt(ed). In the case of a conformal Pt film, electron transfer is more probable as the catalyst surface area is increased, which is also indicated by the much more positive *V*2 value in pn^+^Si/TiO_2_/Pt(sp), shown in ESI Fig. S11.† For example, to reach the same saturation photocurrent, a ∼130 mV overpotential is required for pn^+^Si/TiO_2_/Pt(sp) but ∼200 mV for pn^+^Si/TiO_2_/Pt(ed).

What we have already hypothesized by the performance of the different Pt on FTO is confirmed by the determination of the *J*–Δ*V* curves. At similar saturation photocurrents the *J*–Δ*V* (PV mode) of pn^+^Si/TiO_2_/Pt(sp) is essentially identical with the pn^+^Si/TiO_2_/Pt(ed) (see the green and red dashed curves) while the *J*–*V*1 curve (PEC mode) shows a clearly higher FF for the device with sputtered Pt.

### Cu_2_O/Ga_2_O_3_ junction photocathode

**Fig. 4:**
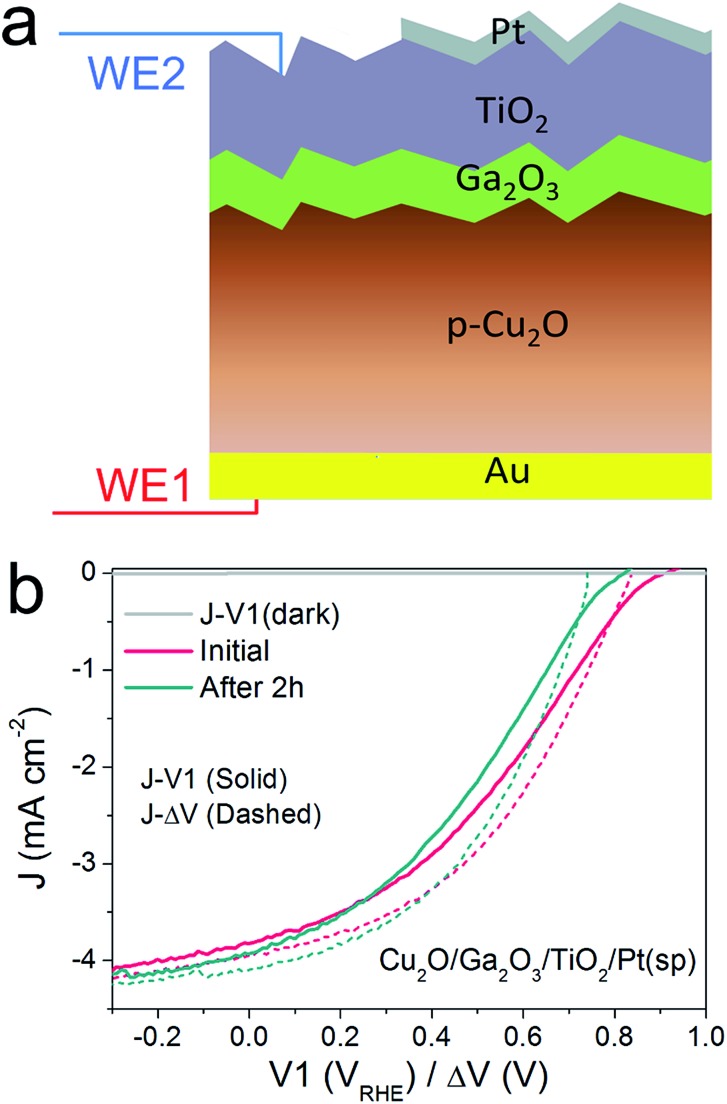
(a) Schematic structure of a Cu_2_O/Ga_2_O_3_/TiO_2_/Pt(sp) photocathode. The thickness of Ga_2_O_3_ and TiO_2_ ALD-layers are 20 and 100 nm, respectively. WE1 controls the back contact potential *V*1 and WE2 measures the surface potential *V*2. (b) *J*–Δ*V* curves (dashed) of Cu_2_O/Ga_2_O_3_/TiO_2_/Pt(sp) before and after 2 h stability test, overlaid with the corresponding *J*–*V*1 curves (solid).

Thus far, we have developed the DWE technique with a model pn^+^Si/TiO_2_/Pt(ed) photocathode, with which we can gain a deeper insight into the PEC process and the photocathode stability. Next, we applied this technique to the emerging material ALD TiO_2_-protected Cu_2_O to demonstrate the generality of the technique and to probe a potential instability of the photovoltaic output of these materials.^22^,^24^ An n-type Ga_2_O_3_ was used as a buffer layer between the Cu_2_O and TiO_2_ overlayer because this interlayer ensures a positively shifted onset potential, compared to that of n-Al:ZnO (AZO) (ESI Fig. S12†).^25^,^26^ Fig. 4a schematically depicts the multilayer structure of the Cu_2_O/Ga_2_O_3_/TiO_2_ photocathode. In a similar fashion as for the silicon photocathodes described previously, a second working electrode was introduced to probe the surface potential *V*2, prior to deposition of the Pt catalyst by sputtering.

PEC measurements were performed in a pH 5 phosphate/sulfate solution. Fig. 4b displays the *J*–*V*1 and *J*–Δ*V* curves. A positive onset potential of ∼0.9 V_RHE_ is observed in the *J*–*V*1 curve, and at *V*1 = 0 V_RHE_, the photocurrent density is 3.9 mA cm^–2^. The onset potential is much more positive than that from both Cu_2_O/ZnO and Cu_2_O/AZO photocathodes, reflecting the larger photovoltage generated by the Cu_2_O/Ga_2_O_3_ junction. In the case of the *J*–Δ*V* curve, the *V*_oc_, *J*_sc_ and fill factor are 836 mV, 4.0 mA cm^–2^ and 36.1%, respectively. Compared with reported Cu_2_O/Ga_2_O_3_ solar cells in the literature, the *V*_oc_ and fill factor values are comparable, but the *J*_sc_ is lower due to light absorption by the Pt catalyst.^26^,^27^ Still, resistance at the TiO_2_/Pt/electrolyte interfaces contribute to the fill factor loss between the *J*–*V*1 and *J*–Δ*V* curves. The fill factor loss is not very significant. As the *J*–Δ*V* curve mirrors the *J*–*V*1 curve, it is clear that the photovoltaic output of the buried Cu_2_O/Ga_2_O_3_ junction is responsible for the shape of the *J*–*V*1 curve of the photocathode, and not the catalytic activity of the Pt catalyst. When using an electrodeposited Pt catalyst the PEC system exhibits a lower fill factor, indicating that the TiO_2_/Pt/electrolyte resistance indeed can also limit *J*–*V*1 performance, as shown in ESI Fig. S15.† We further carried out a stepwise test on the photocathode under illumination. Results and discussion are provided in ESI Fig. S16 and S17† and confirm our statements above.

To study the stability of the Cu_2_O/Ga_2_O_3_/TiO_2_/Pt(sp) photocathode, a 2 h chronoamperometric measurement was performed under illumination at *V*1 = 0 V_RHE_. Fig. 4b shows the comparison of the *J*–*V*1 curves before and after a 2 h stability test. The onset potential shows a negative shift of nearly 120 mV, although the photocurrent density remains similar. Fig. 4b also shows the initial *J*–Δ*V* curve and the one after the 2 h stability test. An obvious decrease in *V*_oc_ is evident, from 836 mV to 743 mV, while the *J*_sc_ shows a slight increase from 4.0 to 4.1 mA cm^–2^ (ESI Table S1†). In contrast to the pn^+^-Si photocathode, the Cu_2_O/Ga_2_O_3_ photocathode shows a degradation of the photovoltaic output of the underlying buried junction. For the silicon system, *J*–Δ*V* remained constant while the *J*–*V*1 changed (Fig. 2). For the Cu_2_O/Ga_2_O_3_ case, *J*–Δ*V* has changed while *J*–*V*1 remains similar (retains a similar photocurrent and fill factor). In order to determine the origin of the degraded photovoltage, a solid-state measurement was carried out using the DWE photocathode in a 2-electrode configuration, directly obtaining photovoltaic *J*–*V* curves. Fig. S18† shows the *J*–*V* characteristics of the Cu_2_O/Ga_2_O_3_/TiO_2_ DWE device before and after a stability measurement that was carried out at short circuit for 2 h, where an obvious *V*_oc_ decrease in a range of 100 mV was observed. Since corrosion through any pinholes in the ALD protective layer can be ruled out in the solid state measurement, we attribute the loss of the PEC performance in this system to an intrinsic problem with the Cu_2_O/Ga_2_O_3_ junction. Further studies are underway to more clearly identify the underlying reason for the instability of this junction.

## Conclusions

We have developed a new configuration of the DWE technique that is able to probe the surface potential of a water splitting photocathode under operation without interfering in the charge transfer processes at the different interfaces in actual water splitting PEC devices. Although we have focused on ALD TiO_2_ in this work due to its widespread use, we expect that our DWE architecture is applicable to other overlayer materials as well, provided they are sufficiently conductive, which is a requirement for PEC electrodes in any case.^28^ This technique has been demonstrated as a universal method to systematically investigate independently the photovoltaic and electrocatalytic properties of catalyst-modified buried junction photocathodes. A pn^+^Si/TiO_2_/Pt photocathode was first fabricated as a platform to model the DWE system. By means of surface potential measurements, the intrinsic properties of the buried p–n junction were studied, and the hidden *J*–*V* curve of a photovoltaic cell was extracted. Additionally, the fill factor loss between *J*–*V*1 and *J*–Δ*V* curves was identified as a parameter that characterizes the TiO_2_/Pt/electrolyte interface, where the morphology of the catalyst plays an important role. Furthermore, the PEC performance degradation mechanism was investigated and discussed. We have demonstrated that the stability of underlying p–n junctions in buried junction photocathodes can be characterized under *operando* conditions. Finally, we applied the DWE technique to a promising emerging system based on Cu_2_O/Ga_2_O_3_, where it was found that the large photovoltage decreases over time. The degradation in the photovoltage with time was also observed in the solid state, ruling out any potential corrosion by the electrolyte. As new material combinations are synthesized for PEC measurements, the DWE electrode technique enables a rapid diagnosis of the cause of degradation in these systems, while also obtaining the PV characteristics of these newly developed junctions without the need to construct separate PV cells.

## Conflicts of interest

There are no conflicts to declare.

## Supplementary Material

Supplementary information

## References

[cit1] Heller A. (1984). Science.

[cit2] Gratzel M. (2001). Nature.

[cit3] Miller E. L. (2015). Energy Environ. Sci..

[cit4] Prévot M. S., Sivula K. (2013). J. Phys. Chem. C.

[cit5] Nakato Y., Egi Y., Hiramoto M., Tsubomura H. (1984). J. Phys. Chem..

[cit6] Boettcher S. W., Warren E. L., Putnam M. C., Santori E. A., Turner-Evans D., Kelzenberg M. D., Walter M. G., McKone J. R., Brunschwig B. S., Atwater H. A., Lewis N. S. (2011). J. Am. Chem. Soc..

[cit7] Huang Q., Ye Z., Xiao X. (2015). J. Mater. Chem. A.

[cit8] Hisatomi T., Kubota J., Domen K. (2014). Chem. Soc. Rev..

[cit9] Zhang Z., Yates J. T. (2012). Chem. Rev..

[cit10] Pham T. A., Ping Y., Galli G. (2017). Nat. Mater..

[cit11] Nakato Y., Ohnishi T., Tsubomura H. (1975). Chem. Lett..

[cit12] Pinson W. E. (1977). Nature.

[cit13] Wilson R., Harris L., Gerstner M. (1977). J. Electrochem. Soc..

[cit14] Lin F., Boettcher S. W. (2013). Nat. Mater..

[cit15] Hodes G., Thompson L., Dubow J., Rajeshwar K. (1983). J. Am. Chem. Soc..

[cit16] White J. R., Fan F.-R. F., Bard A. J. (1985). J. Electrochem. Soc..

[cit17] Seger B., Pedersen T., Laursen A. B., Vesborg P. C. K., Hansen O., Chorkendorff I. (2013). J. Am. Chem. Soc..

[cit18] Gu J., Yan Y., Young J. L., Steirer K. X., Neale N. R., Turner J. a. (2015). Nat. Mater..

[cit19] Septina W., Prabhakar R. R., Wick R., Moehl T., Tilley S. D. (2017). Chem. Mater..

[cit20] Fountaine K. T., Lewerenz H. J., Atwater H. A. (2016). Nat. Commun..

[cit21] Seger B., Tilley D. S., Pedersen T., Vesborg P. C. K., Hansen O., Gratzel M., Chorkendorff I. (2013). RSC Adv..

[cit22] Paracchino A., Laporte V., Sivula K., Gratzel M., Thimsen E. (2011). Nat. Mater..

[cit23] Kemppainen E., Bodin A., Sebok B., Pedersen T., Seger B., Mei B., Bae D., Vesborg P. C. K., Halme J., Hansen O., Lund P. D., Chorkendorff I. (2015). Energy Environ. Sci..

[cit24] Paracchino A., Mathews N., Hisatomi T., Stefik M., Tilley S. D., Graetzel M. (2012). Energy Environ. Sci..

[cit25] Li C., Hisatomi T., Watanabe O., Nakabayashi M., Shibata N., Domen K., Delaunay J.-J. (2015). Energy Environ. Sci..

[cit26] Lee Y. S., Chua D., Brandt R. E., Siah S. C., V Li J., Mailoa J. P., Lee S. W., Gordon R. G., Buonassisi T. (2014). Adv. Mater..

[cit27] Minami T., Nishi Y., Miyata T. (2013). Appl. Phys. Express.

[cit28] Azevedo J., Tilley S. D., Schreier M., Stefik M., Sousa C., Araújo J. P., Mendes A., Grätzel M., Mayer M. T. (2016). Nano Energy.

