# Supplementary material for: Operando deconvolution of photovoltaic and electrocatalytic performance in ALD TiO2 protected water splitting photocathodes
Source: Chem Sci. 2018 Jun 5;9(28):6062–7. doi: 10.1039/c8sc01453a (PMC6052736; doi:10.1039/c8sc01453a)
Supplement: Supplementary file 1 [file SC-009-C8SC01453A-s001.pdf]

Electronic Supplementary Material (ESI) for Chemical Science.
This journal is © The Royal Society of Chemistry 2018

Electronic Supplementary Information

Operando Deconvolution of Photovoltaic and Electrocatalytic Performance in ALD TiO_2 Protected Water Splitting Photocathodes

Wei Cui,[a] *Wenzhe Niu,*[ab] *René Wick-Joliat,*[a] *Thomas Moehl*[a] *and S. David Tilley*[*a]

[a] Department of Chemistry, University of Zurich, Winterthurerstrasse 190, CH-8057 Zurich, Switzerland.

[b] State Key Laboratory of Silicon Materials, School of Materials Science and Engineering, Zhejiang University, Hangzhou, Zhejiang, China.

Experimental Methods

Si wafer cleaning. (111)-oriented Si wafers (thickness ~0.5 mm) used for DWE fabrication were purchased from PrimeWafers. The p-Si substrate was lightly boron-doped (~2×10^{16} cm^{-3}) and a 2 μm-thick n-type surface layer (polished) was doped with phosphorus (~2×10^{19} cm^{-3}). These wafers were cut into 2.5 × 1 cm^2 pieces and sonicated sequentially in acetone, ethanol and MilliQ water (18 MΩ) for 10 minutes each. Next, a two-step deep cleaning was accomplished by using a 5:1:1 mixture of H_2O:NH_4OH:H_2O_2, followed by a 5:1:1 mixture of H_2O:HCl:H_2O_2, both at 50 °C for 10 minutes, in order to completely remove organic and inorganic contaminants. The native oxide layer was etched away by dipping the wafer pieces in 2% HF for 30 s. The samples were then rinsed with deionized water and dried under a stream of nitrogen, and then placed immediately into the ALD chamber for deposition of TiO_2 onto the n^+-Si surface.

Cu_2O plate preparation. A Cu_2O plate was prepared via oxidation of a high purity Cu plate (99.9999%), using a method adapted from the literature.[1] The Cu plate (0.1 mm thick) was first cut into small pieces (~2 × 2 cm^2) and heated from room temperature to 1050 °C (ramp: 17 °C per min) and kept at this temperature for 1 h under Ar flow (1 L min^{-1}). Next, air was introduced into the furnace and held for 3 h. The gas environment was then switched back to Ar for another 3 h of annealing at the same temperature. After cooling down to room-temperature under Ar, the as-prepared Cu_2O plate was dark red when held up to the light.

Atomic layer deposition of TiO_2 and Ga_2O_3. TiO_2 on Si wafer and Ga_2O_3-TiO_2 on Cu_2O plate were deposited by atomic layer deposition (ALD) using a Picosun R200 tool. Before the deposition, the sample was rinsed with deionized water and dried under a stream of N_2. The samples were then placed inside the ALD chamber, which was already heated to 120 °C. Tetrakis(dimethylamino)titanium (Sigma-Aldrich) and H_2O were used as the precursor for Ti and O, respectively. The Ti precursor was heated to 85 °C and a 1.6 s pulse was used (with software boost function), followed by a 6.0 s N_2 purge. H_2O was held at room temperature and a 0.1 s pulse was used, followed by a 6.0 s N_2 purge. To reach 100 nm of thickness for TiO_2, 1860 cycles were used. Measurement of the thickness of ALD-TiO_2 deposited on a piece of Si witness wafer was carried out by ellipsometry (alpha-SE, J.A. Woolam Co.), and fitted with a model for transparent films**.** The cross sectional SEM image (Supplementary Fig. S1) shows that the ALD-TiO_2 protective layer is conformably coated on the Si wafer.

For the Ga_2O_3 layer, bis(μ-dimethylamino)tetrakis-(dimethylamino)digallium (STREM, 98%) was used as Ga precursor. The ALD chamber temperature was kept at 160 °C during deposition. The Ga precursor was held at 150 °C and a 2.5 s pulse was used (with software boost function), followed by a 7.0 s N_2 purge. The H_2O was held at room temperature, and a 0.1 s pulse was used, followed by a 4.0 s N_2 purge time. To deposit a 20 nm-thick Ga_2O_3 thin film, 250 cycles were used. In order to avoid ALD growth on the back side of the sample (potentially leading to shunting problems), teflon tape was used to cover

the back side of the Si wafers and Cu_2O plates during the ALD process. Supplementary Fig. S13 present the $Cu_2O/Ga_2O_3/TiO_2$ multilayered structure.

Fabrication of Si-based DWE. After ALD TiO_2 deposition, the working electrode 1 (WE1) contact was made to the back side of the p-type silicon by scratching the wafer, applying Ga–In eutectic (Aldrich) and attaching copper foil (Aldrich). A layer of epoxy resin (Loctite Epoxide-resin EA 9461) was then used to cover and glue the electrode to a glass microscope slide, with a certain portion of the TiO_2 surface left uncovered for use as the electrochemical active area. For making the front contact, a 20 nm-thick Au layer was sputtered (Safematic CCU-010) onto the epoxy as well as a small part of the exposed TiO_2. A copper wire was connected to the Au layer with Ag paint (Ted Pella, Inc.) on top of the epoxy, as a connection for the second working electrode (WE2). Finally, the front contact was protected from the electrolyte by masking it with a second epoxy layer. Supplementary Fig. S2 shows an optical photograph and the structure scheme of an as-fabricated pn^+Si/TiO_2 DWE.

Fabrication of Cu_2O-based DWE. After the deposition of the Ga_2O_3-TiO_2 overlayer, a 100 nm-thick Au layer was then sputtered onto the back side of the Cu_2O plate (the front side was protected with teflon tape), followed by connecting an Ag wire with Ag paint as WE1. Epoxy resin was then used to cover the whole back side of the electrode to provide protection and enhance the stiffness of the Cu_2O plate. WE2 was connected to the ALD-TiO_2 surface using the same method as for the Si-based DWE, described above.

Platinum catalyst deposition. For some samples, Pt catalyst was deposited onto the as-prepared DWE via galvanostatic electrodeposition from a 1 mM H_2PtCl_6 aqueous solution, denoted as Pt(ed). A constant current of -0.85 μA cm^{-2} was applied to the back contact (WE1) for 15 min. For other samples, nominally 2 nm-thick Pt catalyst was deposited by sputter coating, denoted as Pt(sp).

Characterization. The morphologies of electrodeposited Pt and sputtered 2 nm-thick Pt film, and the cross-sectional scanning electron microscopy (SEM) images of pn$^+$Si/TiO_2 and Cu_2O/Ga_2O_3 photoelectrodes were obtained with a Zeiss Supra 50 VP scanning electron microscope. The polycrystalline structure of the Cu_2O plate is revealed by the X-ray diffraction (XRD) pattern (Supplementary Fig. S14), using a Rigaku Smartlab diffractometer with Cu Kα radiation. UV-VIS spectra of the Pt(ed) and Pt(sp) on FTO slides were recorded with a Shimadzu UV-3600Plus UV/Vis/NIR spectrometer equipped with an integrating sphere.

Photoelectrochemical Measurements. Photoelectrochemical measurements were performed in a four-electrode configuration using a BioLogic SP-300 bipotentiostat. The reference electrode was Ag/AgCl (0.197 V vs. NHE) and a Pt wire served as the counter electrode. All electrode potentials were converted into RHE scale: at room temperature, $V_{RHE} = V_{NHE} + 0.059 \times pH = V_{Ag/AgCl} + 0.059 \times pH + 0.197$. Before the measurements, the electrolyte was sparged with N_2 for at least 10 min to remove dissolved oxygen. Simulated one sun illumination (100 mW cm^{-2}) was provided by a 150 W Xe-lamp with AM 1.5 G filter from LOT Oriel, and the intensity was

calibrated with a standardized silicon diode from PV Measurements (USA). 0.5 M H_2SO_4 and 0.1 M pH 5 phosphate solution (containing 0.5 M Na_2SO_4) were used for Si-based and Cu_2O-based DWE experiments, respectively. Linear sweep voltammograms (LSVs) were collected by sweeping the back contact potential (V1). V1 stepwise measurements were performed by potential step chronoamperometry (CA). Each V1 potential step had a duration of 30 s. The stability tests of the samples were performed for 2 h with V1 held at 0 V_{RHE} under one sun illumination. During all measurements, the second working electrode was kept at open circuit to record the surface potential (V2) against the reference electrode.

Faradaic efficiencies. The Faradaic efficiencies of the photocathodes were measured in a gas-tight three-compartment cell in a three-electrode configuration, with an Ag/AgCl reference electrode and a Pt wire counter electrode. The photocathodes were covered with epoxy to fix the active area to ~0.08 cm^2. The measurement was performed in the same electrolyte as in the PEC measurements (described above). The electrolyte was stirred and constantly sparged with Ar gas at a rate of 20 ml/min. The gas outlet from the cell was connected to a 450-GC Gas Chromatograph Bruker Daltonics GmbH for gas analysis. One LSV scan was first performed for choosing a suitable V1 potential for the Faradaic efficiency tests. During the measurement, the exposed area of the photocathode was illuminated with a white-light LED. The intensity of the light was calibrated to reach a similar photocurrent density as obtained under simulated one sun illumination, as described above

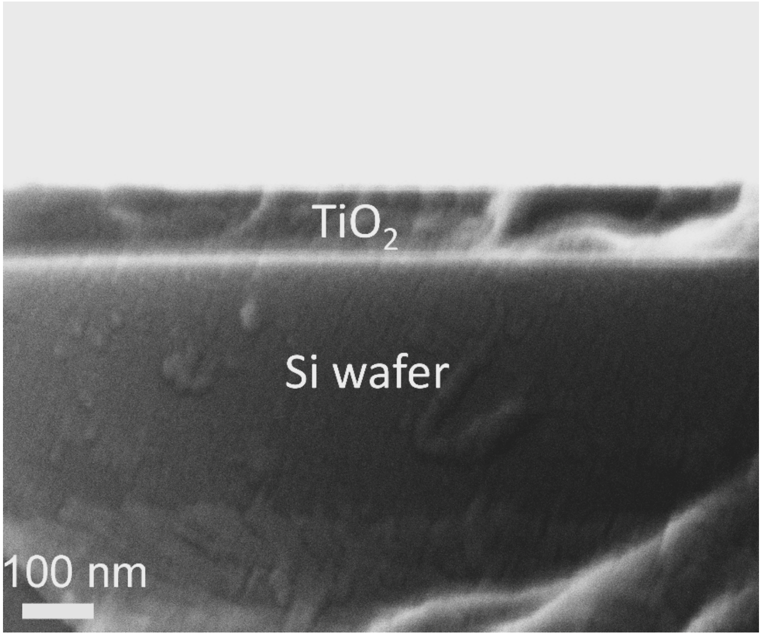

Supplementary Fig. S1 Cross-sectional SEM image of the pn$^+$-Si wafer with 100 nm-thick ALD-TiO_2 protective layer.

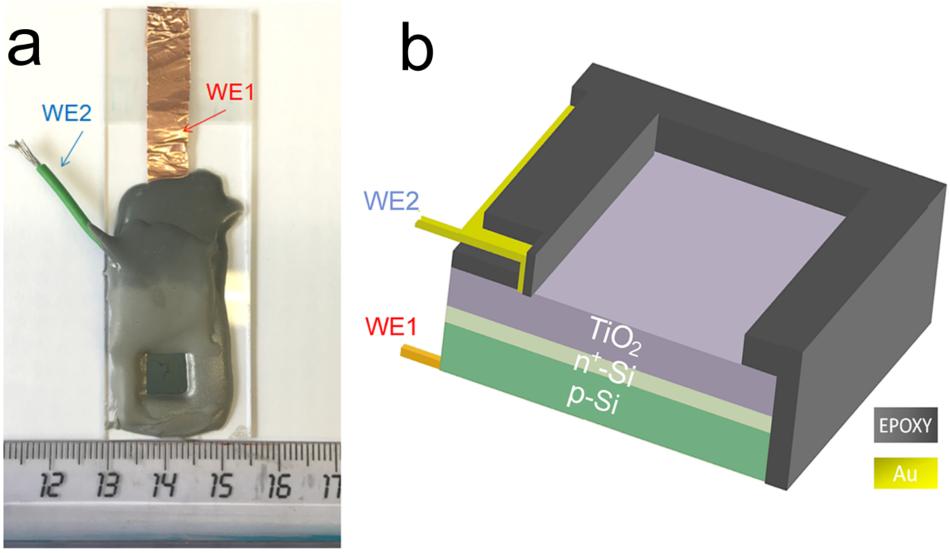

Supplementary Fig. S2 (a) Photograph and (b) structure scheme of a pn$^+$Si/TiO_2 DWE device. The 100 nm-thick ALD-TiO_2 overlayer gives a dark green color on the Si wafer due to optical effects. WE1 is a piece of Cu foil attached to the back side of the p-Si via Ga–In eutectic. WE2 is a conductive cable directly connected to the TiO_2 layer via a 20 nm-thick Au layer and Ag paint. The electrode is protected by epoxy. The ruler is in centimeters.

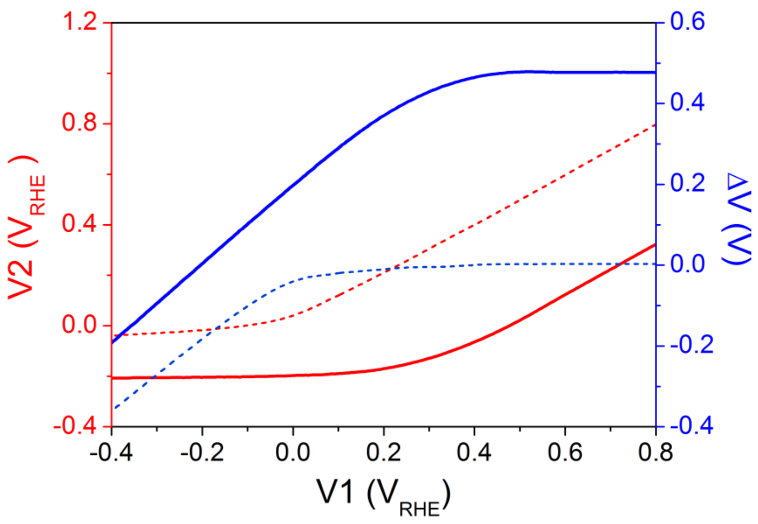

Supplementary Fig. S3 V2 and ΔV as a function as linearly swept V1 in dark (dashed) and light (solid) conditions.

When V1 is more positive than 0 V_{RHE}, V2 is similar to V1, thus ΔV is 0, indicating that no voltage difference is generated under dark conditions in this potential region. When V1 is scanned to more negative potentials than 0 V_{RHE}, V2 stays mainly unchanged with a value slightly more negative than 0 V_{RHE}, contributing to the tiny dark current. This region of very negative V1 corresponds to a reverse bias across the p–n Si homojunction, as is evident from the ΔV-V1 curve.

Under illumination, ΔV maintains a constant value of 475 mV until V1 reaches ~0.47 V_{RHE}. In this positive potential region, ΔV equals the output V_{oc} as there is no current flow across the interface. The potential where ΔV begins to shrink indicates the real onset potential of hydrogen production, which appears in this case at ~0.47 V_{RHE}. As the photocurrent increases while sweeping the potential negatively, ΔV decreases until a saturation photocurrent density is obtained, and then continues to shrink as the photocurrent remains saturated.

J-ΔV		V_{oc} (mV)	J_{sc} (mA cm^{-2})	FF (%)	η (%)
$pn^{+}Si/TiO_2/Pt$(ed)	Initial scan	480	24.9	58.4	6.8
	After 2h	470	24.1	57.7	6.5
	After replatinize	479	24.4	57.6	6.5
$pn^{+}Si/Ti/Pt$(ed)	Initial scan	448	23.5	57.7	6.1
	After 2h	448	22.7	59.7	6.1
$pn^{+}Si/TiO_2/Pt$(sp)	One sun	447	18.2	60.8	5.0
$pn^{+}Si/TiO_2/Pt$(sp)	~ 1.4 sun	473	24.8	60.6	5.2
$Cu_2O/Ga_2O_3/TiO_2/Pt$(sp)	Initial scan	836	4.0	36.1	1.5
	After 2h	743	4.1	44.5	1.3

Supplementary Table S1 J-V parameters extracted from the J-ΔV behavior of the water splitting photocathodes. We expect the pn^{+}-Si junction samples from the same wafer to generate the same V_{oc}. The small V_{oc} variation among $pn^{+}Si/TiO_2/Pt$(ed), $pn^{+}Si/Ti/Pt$(ed), $pn^{+}Si/TiO_2/Pt$(sp) is likely due to the different light intensity as a result of light absorbtion by the catalyst and overlayers, or perhaps passivation and recombination at the Si/overlayer interface (through pinholes). The power conversion efficiency (η) is given for completeness, and is defined as:

$$\eta = \frac{FF \times J_{sc} \times V_{OC}}{P_{in}}$$

where P_{in} of one sun illumination is the incident illumination power density (100 mW cm^{-2}). For the $pn^{+}Si/TiO_2/Pt$(sp) sample, an increased light intensity (P_{in} = ~137 mW cm^{-2}) was also used.

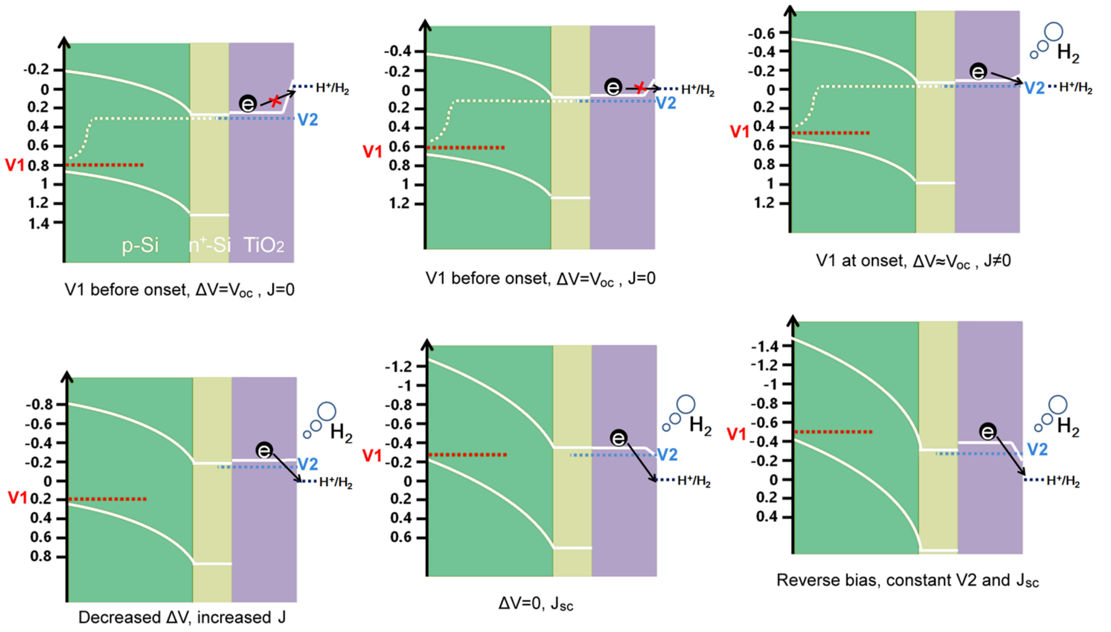

Supplementary Fig. S4 Schematic band energy diagrams of a pn^+-Si/TiO_2 photocathode during a LSV scan by controlling V1 (under illumination). The potentials of the y-axis are V_{RHE}, and $E(H^+/H_2)$ is at 0 V_{RHE}. The Pt catalyst at the TiO_2 surface is not depicted in the schemes. Due to the high carrier doping density in both n^+-Si and TiO_2, the electron (quasi)-Fermi levels are located very close to the conduction band edge, and the space charge region widths are very short, ensuring that Ohmic contacts are formed at both the n^+-Si/TiO_2 and TiO_2/Pt interfaces.

A pn/cat electrode can be seen as a PV cell with one of the current collectors replaced by the electrolyte solution, where under certain conditions of V1, current can flow due to a chemical redox reaction at the surface. Schematically illustrated above, at very positive V1, such as 0.8 or 0.6 V_{RHE}, the electron quasi-fermi level (V2) is still much more positive than $E(H^+/H_2)$. Photoexcited electrons cannot be transferred into solution since the potential of the photoexcited electrons is still not high enough to drive proton reduction. In this region of V1 (before the onset of hydrogen generation), ΔV values remain constant and equal to V_{oc}. The flat band potential of TiO_2 itself is located near $E(H^+/H_2)$. As soon as V2 is slightly more negative than 0 V_{RHE},

hydrogen evolution is thermodynamically allowed, and electrons flow into the electrolyte by reducing protons into hydrogen gas. The V1 potential at this point is defined as the onset potential. As V1 becomes more negative, the ΔV value shrinks as the photocurrent corresponding to hydrogen generation increases. When V1 catches up to V2, ΔV is 0 and the buried p–n junction is at the short-circuit condition. Finally, as soon as the photocurrent becomes saturated due to the photon flux and recombination, V2 reaches a steady value independent of V1. The reverse bias across the p–n junction becomes stronger as V1 level continues to move to more negative potential.

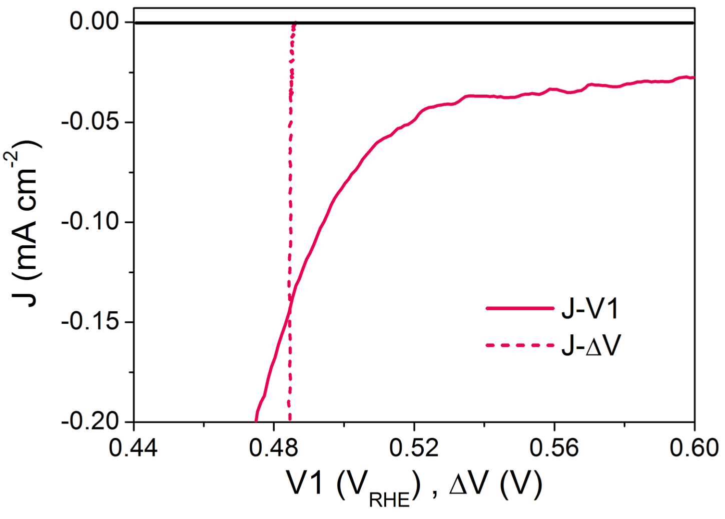

Supplementary Fig. S5 Zoom-in view of J-V1 (solid) and J-ΔV (dashed) curves of a $pn^+Si/TiO_2/Pt(ed)$ photocathode with a scan rate of 10 mV s^{-1}.

The V_{oc} appears at a more negative potential than the onset of photocurrent under certain sweeping conditions, as shown in Supplementary Fig. S5. This means that V2 is more positive than 0 V_{RHE} while current is flowing. In order to confirm that these small photocurrents

correspond to hydrogen evolution and not to e.g. proton intercalation, we carried out faradaic efficiency measurements (See Supplementary Fig. S6). Hydrogen was indeed observed at very small cathodic photocurrent densities, such as -0.08 mA cm^{-2}, at potentials more positive than we would expect from the J-ΔV analysis. This phenomenon likely arises from the fact that the slightly lower electron density than in the illuminated area and consequently an actually slightly reduced V_{oc} is detected compared to the illuminated area (as shown in Fig. 1b). Thus, the onset potential appears earlier than the V_{oc} in Supplementary Fig. S5. This V_{oc} loss is highly dependent on the distance between the Au contact and the illumination area, represented by **d** in this scheme. For all the samples we measured, **d** is ~1 mm. When intentionally lengthening **d**, V_{oc} further decreases, yielding ~75 mV loss at **d** = 3 mm. From our experience with more than fifty samples, the offset between the measured V_{oc} and the apparent onset from the J-V1 curves is typically a few tens of mV with a platinum catalyst.

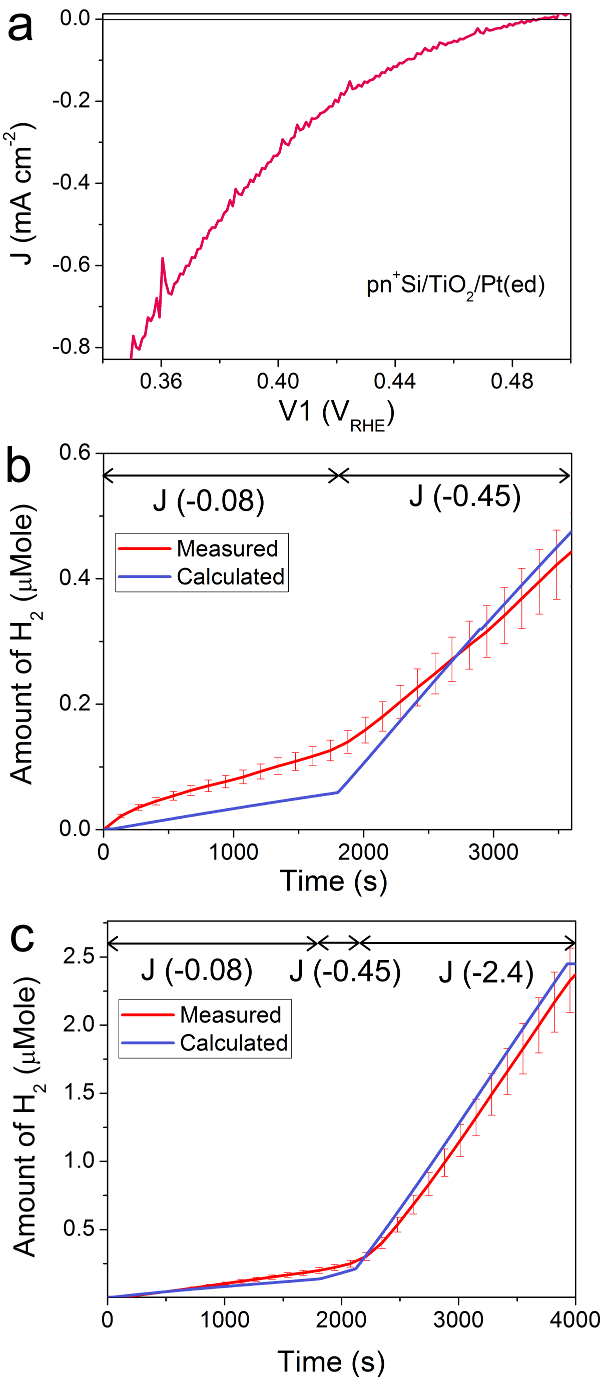

Supplementary Fig. S6 (a) J-V1 curve of a pn$^+$Si/TiO_2/Pt(ed) photocathode for faradaic efficiency measurements. (b)-(c) Comparison of calculated (by photocurrent) and measured H_2 (by GC) under current control, due to the variation of potential with illumination intensity. Note that when the photocurrents are very small, precise measurement of the amount of H_2 by GC is difficult, resulting in seemingly >100% faradaic efficiency for the very small values.

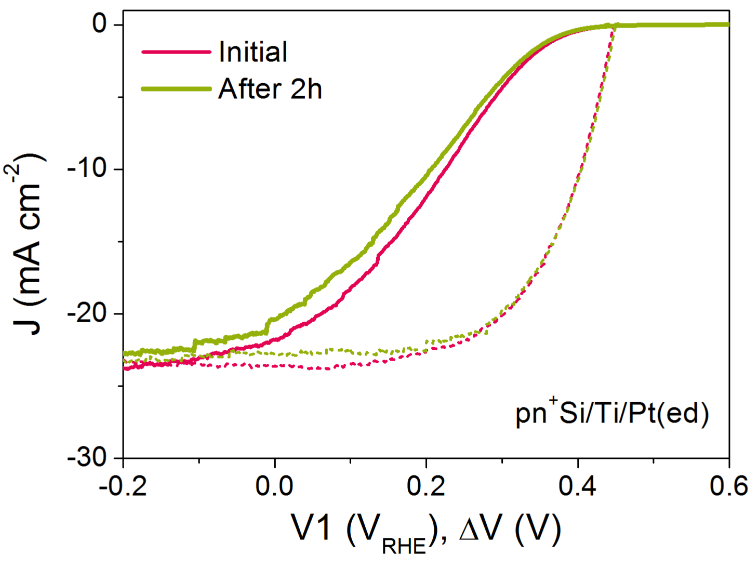

Supplementary Fig. S7 J-V1 (solid) and J-ΔV (dashed) curves of pn$^+$Si/Ti/Pt(ed) before and after 2 h stability test.

A V_{oc} of 448 mV and a J_{sc} of 23.5 mA cm^{-2} are obtained from the initial J-ΔV curve, slightly smaller than that of the pn$^+$Si/TiO_2/Pt(ed) photocathode, which may be due to the antireflective property of TiO_2 layer.[2] The initial J-V1 curve exhibits a large fill factor loss relative to the J-ΔV curve. The similarities of the J-ΔV parameters of this sample with those of the ALD-protected sample indicates that the 100 nm-thick TiO_2 layer is highly conductive and does not contribute to a loss of fill factor in the J-V1 curve. After the 2 h stability test, the saturated photocurrent, V_{oc} and fill factor of the J-ΔV curves remained the same (see Supplementary Table S1), showing that the pn$^+$-Si junction is well protected by the thin Ti layer during the 2 h test.

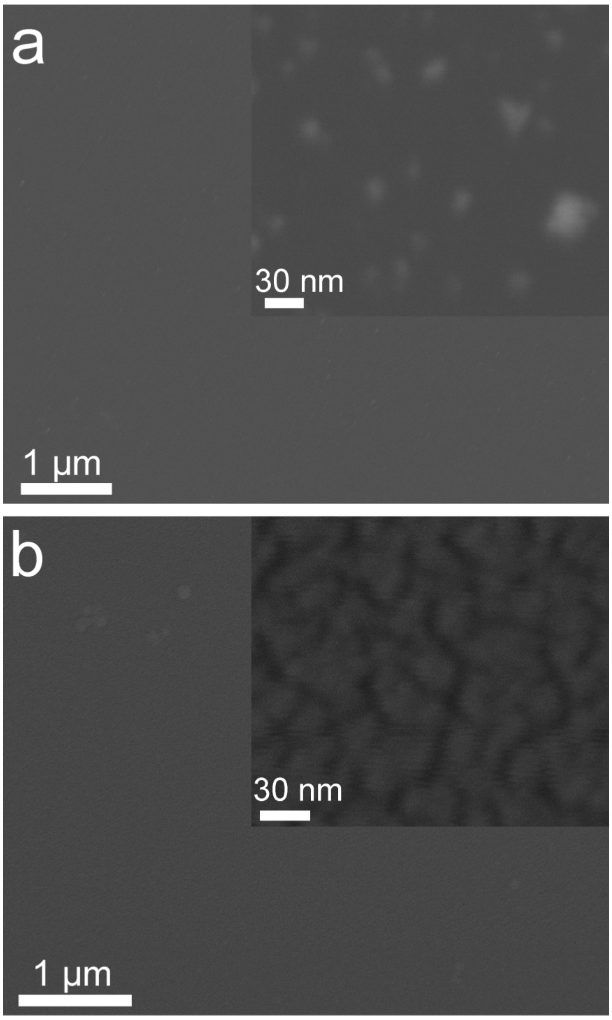

Supplementary Fig. S8 Plan view SEM images of (a) pn$^+$Si/TiO$_2$/pt(ed) and (b) pn$^+$Si/TiO$_2$/pt(sp) photocathode.

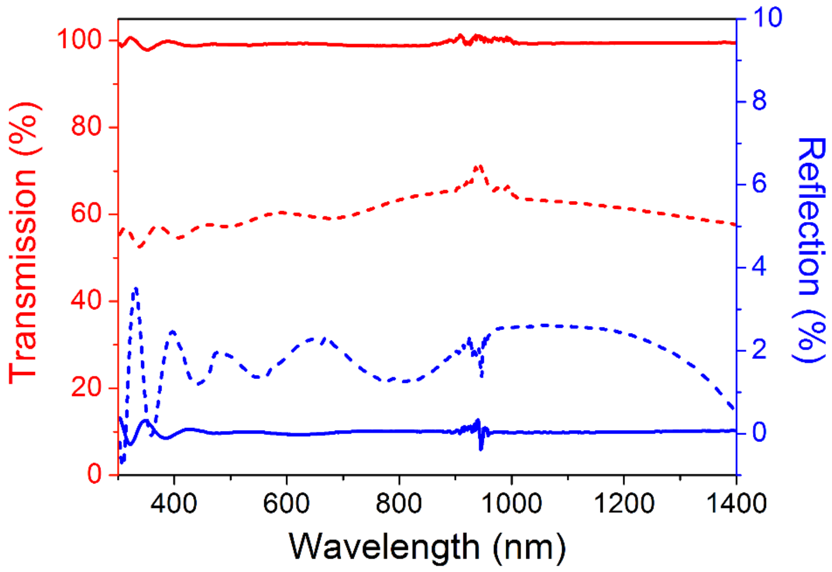

Supplementary Fig. S9 Spectral transmission and reflectance curves for a Pt(ed) (solid) and Pt(sp) (dashed) deposited on FTO slides.

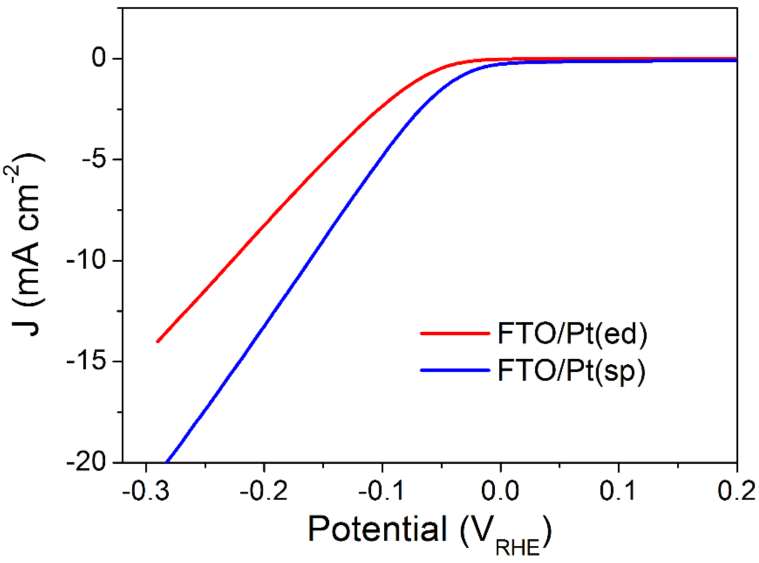

Supplementary Fig. S10 J-V curves of Pt(ed) and Pt(sp) deposited on FTO slides in 0.5 M H_2SO_4.

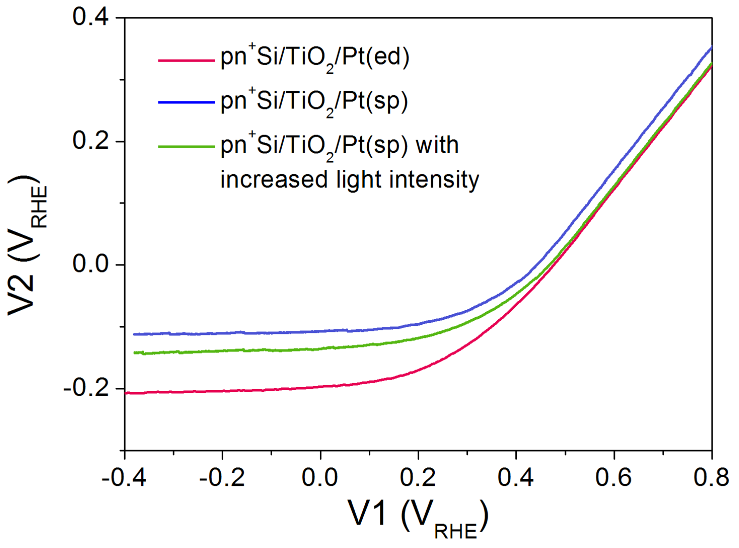

Supplementary Fig. S11 V2-V1 curves of a pn$^+$Si/TiO_2/Pt(ed) and a pn$^+$Si/TiO_2/Pt(sp) photocathodes (under one sun illumination). For comparison, the performance of pn$^+$Si/TiO_2/Pt(sp) with similar photocurrent densities as pn$^+$Si/TiO_2/Pt(ed), by increasing the light intensity, is also displayed (the green curve).

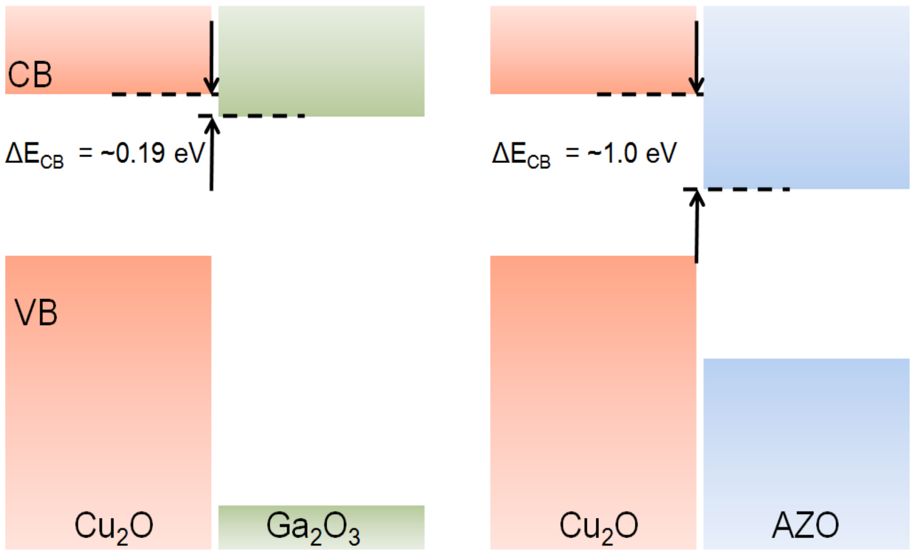

Supplementary Fig. S12 Schematic band alignment of the Cu_2O/Ga_2O_3 and Cu_2O/AZO heterojunctions. Cu_2O/Ga_2O_3 exhibits a small conduction band offset (ΔE_{CB}) of ~0.19 eV. This value is much smaller than the counterpart of Cu_2O/AZO (~1 eV). Therefore, compared with AZO, Ga_2O_3 shows an improved conduction-band offset with Cu_2O, enabling a larger photovoltage and the ability to shift the onset potential positively.

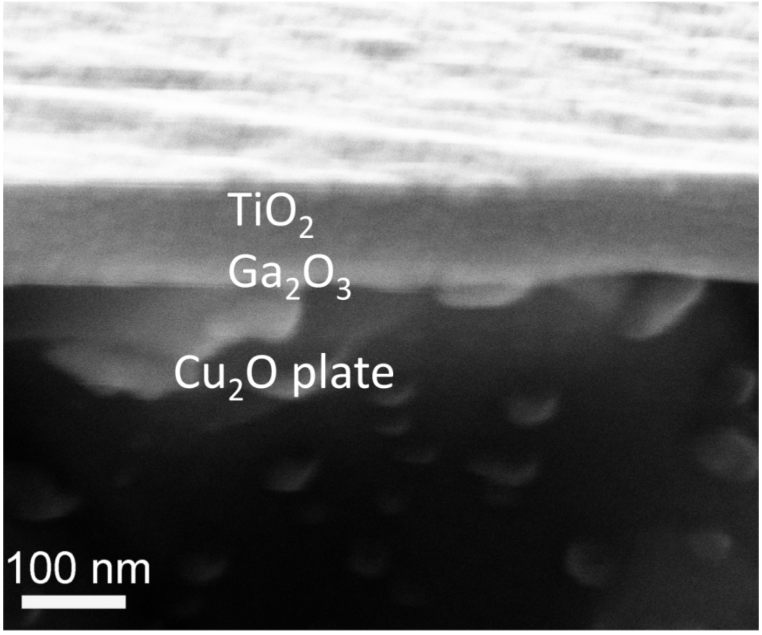

Supplementary Fig. S13 Cross-sectional SEM image of $Cu_2O/Ga_2O_3/TiO_2$ electrode. The thickness of Ga_2O_3 and TiO_2 are 20 nm and 100 nm, respectively.

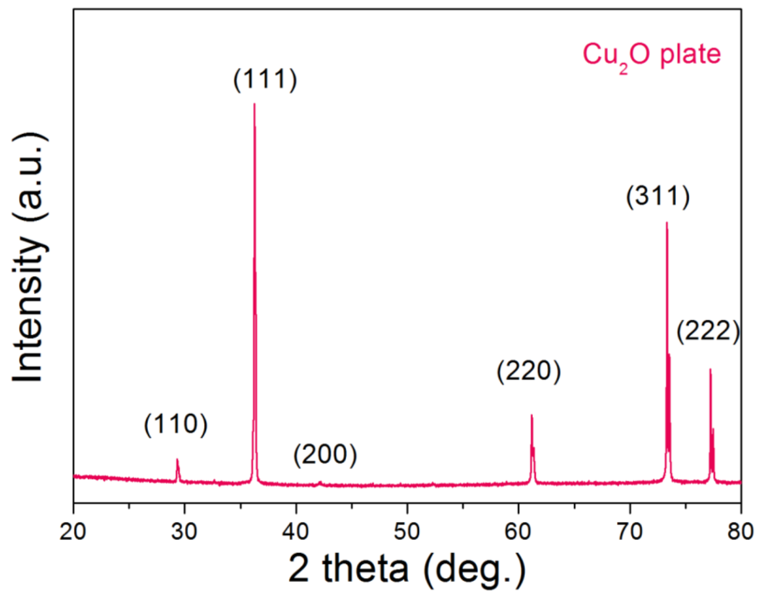

Supplementary Fig. S14 The XRD pattern of the synthesized Cu_2O plate. Identification of the diffraction peaks is referred to the standard Cu_2O data (JCPDS No. 05–0667).

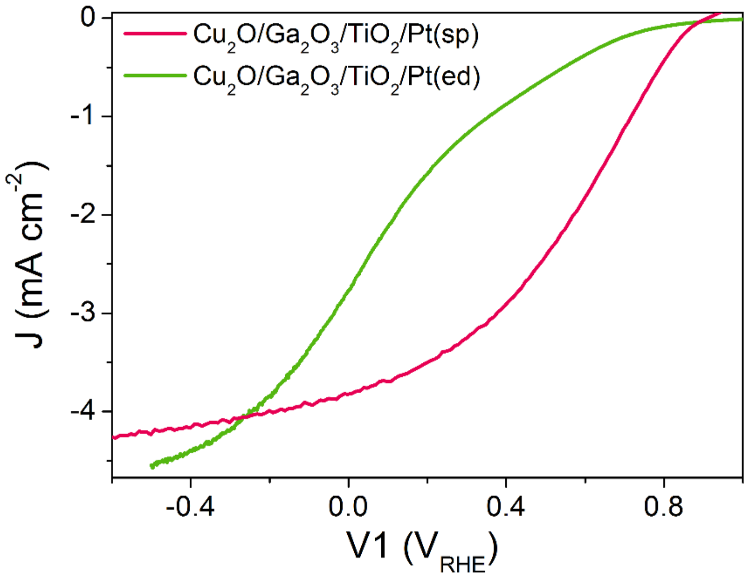

Supplementary Fig. S 15 J-V1 curves of a $Cu_2O/Ga_2O_3/TiO_2$/Pt(sp) and a $Cu_2O/Ga_2O_3/TiO_2$/Pt(ed) photocathodes (under one sun illumination).

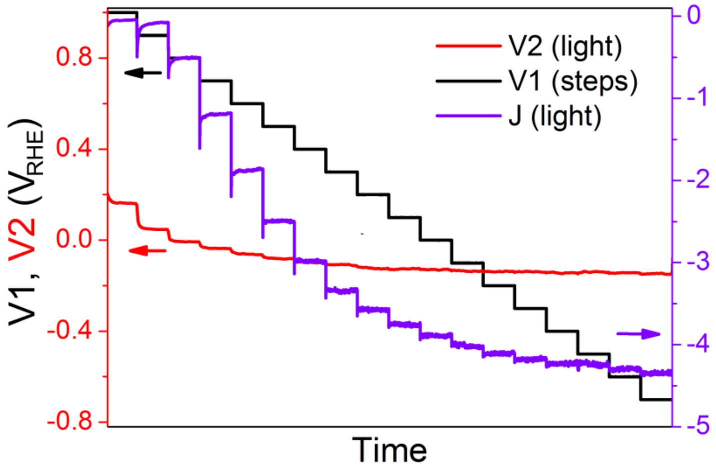

Supplementary Fig. S16 V2 and J values of the $Cu_2O/Ga_2O_3/TiO_2$/Pt(sp) photocathode under stepwise controlled V1 under one sun illumination. Each V1 step lasts 30 s.

When V1 is more positive than 0.8 V_{RHE}, V2 is more positive than 0 V_{RHE} but accompanied by a small photocurrent density. In this region, ΔV is determined as ~800 mV. When V1 is more negative than 0.8 V_{RHE}, the photocurrent increases and ΔV shrinks dramatically, following the same trend as with the pn^+/Si photocathode. Eventually the photocurrent reaches a plateau and V2 likewise saturates, even as the V1 potential moves steadily more negative.

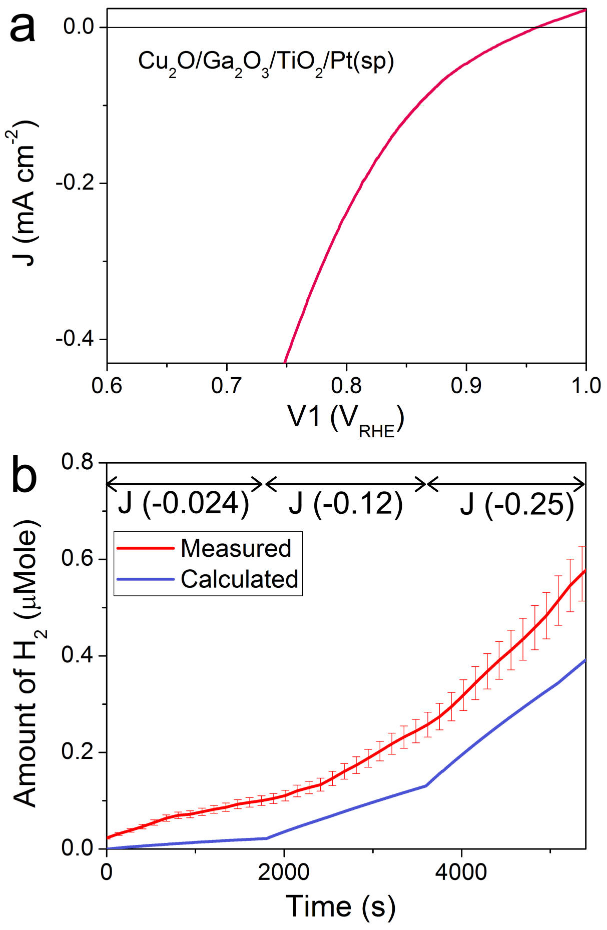

Supplementary Fig. S17 J-V1 curve of a $Cu_2O/Ga_2O_3/TiO_2$/Pt(sp) photocathode for faradaic efficiency measurements. (b) Comparison of calculated (by photocurrent) and measured (by GC) H_2 under current control, due to the variation of potential with illumination intensity. Note that when the photocurrents are very small, precise measurement of the amount of H_2 by GC is difficult, resulting in seemingly >100% faradaic efficiency for the very small values.

As seen also with the silicon-based photocathode, the onset potential occurs earlier than the V_{oc} (Fig. 4b). Again, faradaic efficiency measurements were carried out at very small photocurrent densities, (e.g. -0.024 mA cm^{-2}), which confirmed that these currents do indeed correspond to hydrogen evolution. The rationale for the discrepancy between the apparent onset and the measured V_{oc} is the same as for the silicon-based photocathode, discussed in Supplementary Fig. S7.

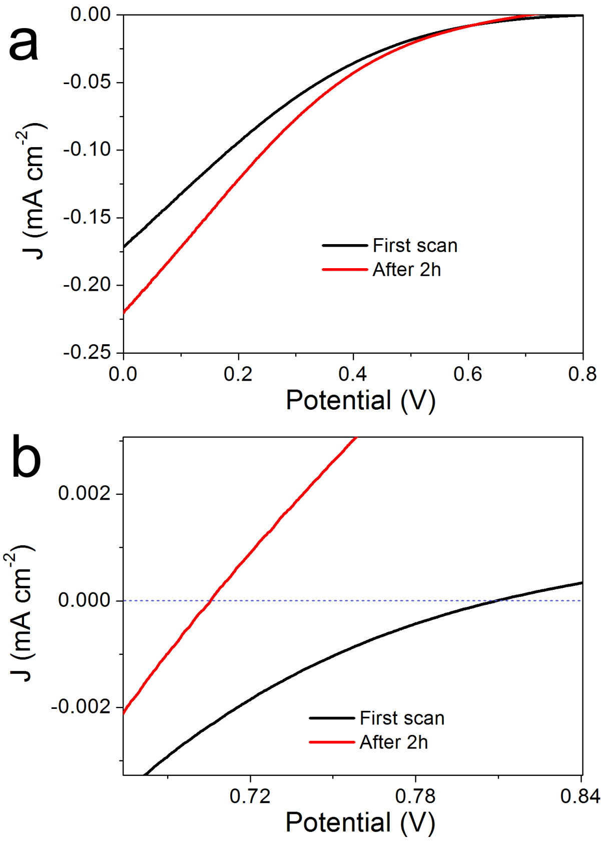

Supplementary Figure S18 (a) Solid-state J-V curves of a $Cu_2O/Ga_2O_3/TiO_2$ DWE measured in 2-electrode configuration (in air, no electrolyte present), before and after a 2 hour stability measurement at short circuit under one sun illumination. We note that the fill factor is poor and the series resistance is high since our DWE architecture was designed only to sense the potential of the surface with current being extracted into the electrolyte, and not to efficiently extract current through WE2. (b) Zoom-in of the V_{OC} region.

REFERENCES

(1) Minami, T.; Nishi, Y.; Miyata, T.; Nomoto, J. High-Efficiency Oxide Solar Cells with ZnO/Cu2O Heterojunction Fabricated on Thermally Oxidized Cu2O Sheets. *Appl. Phys. Express* **2011**, *4* (6), 62301 DOI: 10.1143/apex.4.062301.

(2) Richards, B. S. Comparison of TiO2 and other dielectric coatings for

buried-contact solar cells: a review. *Prog. Photovoltaics* **2004**, *12* (4), 253–281 DOI: 10.1002/pip.529.
